# The facilitators and impediment factors of structural empowerment in pregnancy and delivery care: achievement of power

**Published:** 2015

**Authors:** M Janighorban, H Yousefi, N Yamani

**Affiliations:** * Department of Midwifery, Nursing and Midwifery Care Research Center, Faculty of Nursing and Midwifery, Isfahan University of Medical Sciences, Isfahan, Iran; ** Department of Adult Nursing, Nursing and Midwifery Care Research Center, Faculty of Nursing and Midwifery, Isfahan University of Medical Sciences, Isfahan, Iran; *** Department of Medical Education, Medical Education Research Center, Isfahan University of Medical Sciences, Isfahan, Iran

**Keywords:** empowerment, pregnancy and delivery care, Midwifery Education, qualitative study

## Abstract

**Background:** The organizations essentially affect empowerment of personnel through the preparation of the needed grounds for them. Also, the students may acquire the required potentials and capabilities in the educational organizations when the possibility is provided to them to access power and opportunity in educational environments.

**Objective:** The present study aimed to explain the facilitators and impediment factors of structural empowerment in pregnancy and delivery care.

**Methods:** According to Kanter’s theory, this qualitative study was conducted with the participation of 15 superior midwifery students, ten academic teachers of midwifery, and two midwives employed in the educational hospitals. Data were collected by semi-structured interviews individually and in the group and analyzed by using a directed content analysis method.

**Results:** To explain the facilitators and impediment factors of empowerment in pregnancy and delivery care in the power structure, the access was provided to a support formed by three broad categories of support from the instructors, support from personnel, and support from a classmate. The access to resources was created with three broad categories of access to the appropriate clinical environment, to the laboratory of clinical skills and to information sources, and to information, forming with two broad categories of awareness of the educational objectives as well as legal and legitimate issues.

**Conclusion:** One could prepare the ground for the midwifery students to access this empowerment in pregnancy and delivery cares more than ever by providing equipped clinical environments and the presence of all-inclusive supportive climate in such environments. Along with the efficient training of students in the laboratory

## Introduction

Almost 800 women die due to the relevant adverse effects of pregnancy and delivery throughout the world every day while most of these mortality cases may be prevented [**1**]. The poor quality care for the pregnant women has not led only to the mortality of mothers. One woman out of 760 cases is involved in near-miss morbidity or mortality upon period of delivery in USA [**2**]. The death of the mother will exert destructive effects on the development of countries, the economy in society, family, and emotional and physical adverse effects on children. The negative physical and psychological consequences of the relevant serious complications of pregnancy and delivery will also remain in the surviving mothers for an extended period [**3**-**5**]. Improving health in mothers is deemed as one of the key preferences in the World Health Organization (WHO). Therefore, the execution of the related care by the skilled personnel may play an essential role [**6**]. The management of all the deliveries by the qualified staff is also an indicator that was still put on the international agenda in the sustainable development plan of the millennium after 2015 [**7**]. The midwives are regarded as key a personnel concerning the health of mothers and the newborns [**8**,**9**]. Thus, the presentation of professional education is necessary for the improvement of the coverage and the quality of the given services by midwives [**10**]. However, the studies indicated that modern and interactive approaches have been taken less in education. The midwifery students are exposed to the restricted prime opportunities to acquire the minimum experiences in clinical work, and the acquisition of competency to work in the midwifery profession is not comprehensively and appropriately evaluated [**11**]. Similarly, training and assessment of cognitive skills such as clinical reasoning, problem solving, and critical thinking have not been efficiently incorporated in the curricula for midwifery students, and they do not address [**12**]. 

A review on the status of clinical education in Iran also showed that several barriers might lead to a lack of providing the needed context for students to access favorably empowerment at the professional level. Some of these obstacles are as the following: inappropriate clinical environment [**13**-**15**], non-compliance of routine plan of hospitals and healthcare centers with the curricula and the personnel-related problems [**14**,**16**,**17**], inadequate educational cases of patients in the medical wards for learning [**18**], the problems relating to educational planning [**13**,**18**], weak inter-professional collaborations [**14**,**19**], and the relevant issues to the clinical evaluation of the tools and processes [**18**,**19**]. 

The empowerment of women and their families is the context for midwifery work. Thus, midwives should personally experience this empowerment [**20**]. Kanter argues that those environments may prepare the ground for the empowerment of the personnel who has possible access to information, support, and needed resources as well opportunity for growth and gaining knowledge and skill for doing this job. Support, information, and resources form power structure in an organization [**21**]. Support refers to feedback, guidance, and leading that the individual receives from superiors, peers, and subordinates. The information includes data, technical knowledge, and the necessary expertise, and general information regarding the organization for an efficient performance in the occupational position. Materials, fund, facilities, equipment, needed time for the fulfillment of organizational objectives, form the resources. On the one hand, the preparation of the possible access to power and opportunity structures in the organization have improved motive, job satisfaction, sense of self-efficacy, sense of independence and higher organizational commitment in an individual. And, on the other hand, it is followed by the strengthening of feelings of power, tendency to group synergy, and granting more freedom of action to subordinates [**22**,**23**]. Taking several steps forward in the current situation within the field of healthcare for mothers and neonates, the reduced numbers of caesarean delivery, achievement of internationally agreed goals regarding mortality of mothers will be subjected to the presence of competent and capable personnel in this field. Thus, the present study was carried out based on Kanter’s theory and by aiming at deeply identifying the facilitators and impediment factors for the structural empowerment in pregnancy and delivery care according to the attitude of midwifery students and academic teachers as well as the employed midwives. 

## Study method 

The current study was a qualitative research that was implemented by using a directed content analysis method. The participants were excellent students in midwifery, continuous BS course, midwifery academic teachers, and the employed midwives in the educational hospitals, who were chosen by using a purposive sampling method. The maximum variation considered in selecting the academic teachers was regarding age, working background, educational experience, and educational status and in students regarding empowerment in pregnancy and delivery care (weak, fair, and firm). The employed midwives were also chosen from two main different hospitals for the education of pregnancy and obstetric cares for midwifery students. To conduct this study, initially, this issue was informed to the excellent students in a student meeting and then the researcher interviewed the participants after attending an educating center (hospital, clinic, and faculty) and took an agreement and consent from them. Moreover, teachers and midwives were also interviewed at their workplace. Nine midwifery students were individually interviewed to increase the depth and richness of the data by the aid of group interactions; 6 midwifery students were also interviewed in a group. Ten teachers and two employed midwives were individually interviewed as well. The interviews were executed by one of the researchers, who was the engaged midwifery instructor in the faculty with a MSc. degree in midwifery and as student in the reproductive health doctorate course. The period of interviews varied from 36 to 92 min. The interviews were performed in a semi-structured form. The sampling process ended when no new information was found in interviews and with the saturation of data. Initially, the interview was started with an open-ended question. These issues included the following: How did you evaluate your own in doing pregnancy and obstetric care? What factors have been active in the acquisition of empowerment of pregnancy and obstetric care? (Students) How did the empowerment student operate in pregnancy and obstetric care and which elements were useful in the acquisition of the empowerment of midwifery students in these cares? (Teachers and midwives) Then the interview was continued by persistent questions about the already predicted categories according to Kanter’s theory. The voice of participants was recorded on the voice recorder. After conducting any interview, the recorded voice was transcribed word-by-word by the researcher by using Word processor software and simultaneously the data were analyzed by a directed content analysis method. To immerse in data, an interview text was reviewed and reread for several times. Based on Kanter’s structural theory, opportunity and power structures were employed as primary categories and onset framework. Operational definitions of any group were extracted by using the method and on definitions and aspects of above, the initial codes were obtained. The similar systems were merged and put together in the same category. As a result, the initial groups were formed. These groups were joined again, and they created the final classes. The one-note software was used for the ease of saving, classification, and searching for codes. To ensure precision and rigor of findings, the following strategies were used. In parallel with improving the credibility of the study, participants were chosen with maximum diversity and various techniques were employed for data collection. The accuracy of the data and the extracted codes were revised by the members, and they were corrected if necessary. Data were examined by the supervisor teachers, a person with a PhD degree in curriculum planning and people with a nursing PhD degree to ensure the compliance of the categories with notations of the participants, accurate information of sub-categories within framework and definition of predetermined types of theory and also the rejection of ideas and presuppositions. Data were continuously studied and explored in the course of improving dependability. The activities relating to the quality of data collection and analysis were entirely and consistently recorded. Some examples of the quality of extraction of the meaning units and codes from the body of interviews were presented to the external observer teachers for each of categories. To increase transferability, the given results were presented to some of the participants with specifications of members, who did not attend this study and their judgment was evaluated regarding the presence of similarity between the results of the research and their experiences. In parallel with rising confirmability, texts of some of the interviews, extracted codes, and categories were given to the colleagues of the researcher who were familiar with the method of analysis on qualitative researchers but did not participate in this study and they agreed on the given results. This study was approved by the ethics committee of Isfahan University of Medical Sciences under Code No 393373. The students and other persons were ensured that their remarks would remain confidential. Also during the execution of the work, other ethical considerations were correctly observed. This study was conducted in the faculty of nursing and midwifery at Isfahan University of Medical Sciences. This possibility was provided for a student in this college to study in 4-years midwifery continuous BSc. course, MSc. course, and doctorate level. 

**Findings**

15 students of ages 22-29 participated in this study; they were entirely females, the majority being single ones. The participating academic teachers were between the ages 29 and 53, with 2-26 years working experience and educational records and MSc. course of midwifery or doctorate in reproductive health. The member midwives were 34 years old, with 8 and ten years work background and BS degree in midwifery (**Table 1**,**2**). The current article presents the results in power structure based on two structures of opportunity and power. In order to explain the facilitators and impediment factors of empowerment in pregnancy and delivery care in the authority structure, access to support was formed with 3 main categories of support from the instructors, from personnel and from classmate, access to resources was built with 3 major classes of access to the appropriate clinical environment, access to laboratory of clinical skills and access to information sources, and access to information was created with 2 main categories of awareness of educational objectives, as well as legal and legitimate issues (**Table 3**). The review on the empowerment for pregnancy and delivery care and its active factors in this study represented the presence of a spectrum in this field so that any factor had a positive and facilitating role at one end of this spectrum and on the other hand it had a negative and impediment role at the other end, and, depending on their personal experiences, it referred to both sides of this spectrum in the statements of the participants.

**Table 1:** Personal characteristics of participating students

participant	Marital status	age	occupation
P1	single	22	student
p 2	single	23	student
p 3	married	23	student
p 4	single	22	student
p 5	single	22	student
p 6	single	23	student
p 7	married	22	student
p 8	single	22	student
p 9	single	23	student
p 10	single	29	student
p 11	single	23	student
p 12	single	22	student
p 13	single	23	student
p 14	single	26	student
p 15	single	25	student

**Table 2:** Personal characteristics of participating instructors and midwives

participant	age	occupation instructor/ Midwife	Education	Educational background	working background
p 16	49	instructor	MSc	11	25
p 17	32	instructor	MSc	2	2
p 18	43	instructor	MSc	18/ 5	18/ 5
p 19	29	instructor	MSc	3	3
p 20	41	instructor	MSc	5	14
p 21	53	instructor	MSc	26	26
p 22	34	midwife	BSc	-	8
p 23	49	instructor	MSc	22	22
p 24	34	instructor	MSc	9	9
p 25	48	instructor	PhD	24	24
p 26	34	midwife	BSc	-	10
p 27	43	instructor	PhD	11	19

**Table 3:** Facilitator and impediment factors of empowerment

Facilitator factors	impediment factors
*Support*	
Support from instructor	
Presence of instructor or mentor in clinical environment	Absence of instructor
Educational competency of instructor	Educational incompetency of instructor
Instructor’s positive interpersonal relations	Instructor’s negative interpersonal relations
Professional competency of instructor	Professional incompetency of instructor
Support form personnel	
Occupational competency of personnel	Occupational incompetency of personnel
Personnel’s positive interpersonal relations	Personnel’s negative interpersonal relations
Support from classmate	
Learning to work beside classmate	Lack of synergy
*Resources *	
Access to appropriate clinical environment	
Presence in clinical environment	Clinical environment inappropriate conditions
Access to laboratory of clinical skills	
Efficient training in clinical skills laboratory	Poor training in clinical skills laboratory
Access to information resources	
Ease of access to numerous sources	Difficult access
Awareness of educational goals	
The educational objectives as guide to work in clinical sites	Overlooking educational goals
Awareness of legal and legitimate issues	
Awareness of occupational legal and legitimate issues	Lack of knowledge about the laws

**Support-support from instructor**

Receiving guidance, feedback, and support from professors were assumed as useful factors to acquire empowerment for pregnancy and delivery care in clinical environments. The presence of an instructor or educational mentor in a clinical environment, positive personality traits of the instructor, educational competency of the instructor, positive interpersonal relations of the instructor, and professional competency of instructor formed this major category as the useful factors on empowerment. 

One of the foremost factors in the acquisition of empowerment for pregnancy and delivery care was the fixed presence of the expert instructor in clinical environments. The presence of support of teacher from a student in work and problems acted as a facilitator for the acquisition of self-confidence, better learning, and capability. “If a teacher attends in the site I feel that I can refer to her quickly if any problem takes place. I have more self-confidence and ability because there is someone who may support me”. (Participant student) However, the absence of the instructor or mentor may reduce the educational and the supportive level for a student in a clinical environment. 

The positive personality traits of instructors were considered the following: well morality, having the sense of humor, positive energy, self-confidence, comfort, responsibility, sympathy, accountability. Also, having sense of goodwill in the training of the student was considered as an active factor in creating a peaceful, friendly, positive, and supportive climate in the clinical environment. “Personality is very important. As you see, an instructor displays goodwill and works for you while she does not like to contempt you and she intends to train you can feel sense of comfort and support”. (Participant student) The formation of this peaceful and supportive climate in a clinical environment is due to the positive personality traits of the instructor that may prepare the acquisition of self-confidence, coping with fear, access to further clinical opportunities, better learning, and training for empowerment in the student. The absence of these personality traits in instructors were also deemed as an impediment factor in the access to empowerment per se. 

The competent instructors play an active role in training to compel the students to think, reason and make an effort for learning them. They may play an essential role by taking efficient teaching methods in the clinical field, improving motive in students, and giving the opportunity to develop their capabilities in the clinical environment. “The student sometimes does not know what to do in the clinical environment and me as an instructor should stimulate her while there is some case but the student does not notice them. Creating that question mark in her mind and motivating her is my task as the instructor”. (Participant teacher) However, the instructors’ weakness in compelling the student to make an effort to learn and their failure in conveying knowledge and experience to the student is an impediment factor in the achievement of the student for empowerment. 

The friendly and suitable behavior of the teacher with both the personnel and the student plays an efficient role in the student’s success of empowerment. Attaching respect for the student and identifying her and addressing her requirements upon doing the task along with support and proposing feedback may develop her potential for decision-making and self-confidence in the student. The dominance of enforcement in the teacher’s behavior instead of punishment and also empathy with a student against her fear from error and mistakes causes a reducing of stress, preservation of mental calmness and self-confidence in student and prepares the ground for her capability. 

“I tell the student if you start working for delivery I support you and be sure. If you make any mistake, I will remind you peacefully in such a way that you never be ashamed before others and publically and not to be excited as well. In this way, the student may feel the sense of self-confidence”. (Participant teacher) However, an inappropriate behavior versus the student’s mistakes may eliminate the confidence sense in a patient to her student giving care and also removes self-confidence in a student and it leads to a disruption of the mental comfort, sense of inferiority, inadequacy, and lack of professional capability, that is a barrier against a student on the path toward empowerment. 

The professional skills of instructors are also deemed as an active factor in reaching students to empowerment. The instructors’ interest in the clinical environment and care for the patient as well as having motive and interest in the profession may act as a factor to create interest in students and purpose for learning and empowerment in pregnancy and delivery care in them. “I receive motivation from these points that the teacher has personally motive and is interested in her field of study and from the behavior of instructor with the receiver of care when she gives information to the student. All of these cases reveal teacher’s interest that if she is interested in what she says or not and the instructor conveys this sense to her students”. (Participant student) 

Professional acceptability in a clinical environment, experience, and skill in clinical work, and scientific potential also prepare the ground for a more efficient education and giving further opportunity to students in doing the task and provides further support from students and it acts as a facilitator in the access to their empowerment. In contrast, the lack of proficiency of the teacher in the clinical work and the absence of interest and motive are deemed as an impediment factor against the student’s access to empowerment. 

**Support from personnel **

Staff plays an active role in supporting the midwifery students to acquire empowerment in pregnancy and delivery care. The professional competency of staff and the positive interpersonal relations of personnel have formed this main category. 

If the team, who works in hospitals and the educational healthcare centers, possessed an appropriate scientific and practical potential, it may play an efficient role in the support and empowerment in students. These favorable patterns may play a decisive role in improving motive and making an effort for learning and empowerment of student. “The students will be more enthusiastic if personnel are more well-trained and skillful and with further experiences”. (Participant student) In contrast, the students may be subjected to paradox if they receive erroneous and empirical information from the weak and unskilled personnel and they may learn improper attitudes and take non-scientific measures and thereby their spirit and motive will be weakened for empowerment. 

Positive interactions between the staff and students are also considered as a facilitator factor to the access to empowerment. The team plays a decisive role in its progress through conveying experiences, assignment of a task, guidance, training, and support during working and proposing feedback to the student. In contrast, fewer interactions between the personnel and students and the lack of their motive in communication with students on the one hand, and negative statement and behavior and inappropriate expectation from the student, exertion of discrimination among midwifery students with medicine students act as an impediment factor against a student from making an effort for empowerment. “Sometimes, personnel may reduce sense of self-confidence in students since they have learned some issues by experience that might not be fundamental and therefore they expect from you to implement the same principles in work, indeed if you do not implement it they will treat unfavorably with you”. (Participant student) 

**Support from classmate**

The assistance and support of classmates from each other upon doing a task is one of the decisive factors in the achievement of empowerment. Listening to strength and weakness points of the function from other classmates has led to the improving trend of doing work and developing individual capabilities. “Simply work of IV catheterizing may be stressful for you if you like doing it alone. I gave help to my friend and she also gave aid to me. Then we supported from each other. Now, the teacher is absent to see our weak point, but the classmate is present to tell us which task did not conduct properly”. (Participant student) 

Jealousy in some classmates in expressing the realities of doing work and the adverse reaction from some others to show their mistakes by friends are an impediment factor for the synergy with classmates, in learning the work and acquisition of empowerment. 

**Resources**

**Access to appropriate clinical environment**

Access to appropriate clinical conditions deemed as a source of practical factors in the purchase of empowerment for pregnancy and delivery care. Presence in real clinical environments and its continuity may provide an opportunity for using individual potentials, doing work independently, acquisition of self-confidence, and capability. After attendance of actual conditions of clinical application, individuals perceive theoretical lessons, and they can achieve retained and efficient learning. Observation of high points of their work in comparison with other students and personnel paves the way for the acquisition of self-confidence further and empowerment in work. “If I compare myself with other staff I am not lower than them not at all because I went and saw how they were working”. (Participant student) 

Inappropriate facilities and environmental conditions may prepare the ground for incapability in doing skill correctly and entirely and reduced learning and act as a barrier against a student to access to empowerment. 

**Access to laboratories of clinical skills**

The clinical skill laboratories have an unusual position in empowerment for pregnancy and delivery care. This environment is a place for the implementation of theory into practice and embodiment of real clinical environment. “It is very favorable for the student to become familiar with practical and more tangible cases. It is a place separated from the hospital that you can touch it. It was favorable that you could exercise IM injection and IV catheterization”. (Participant student) 

In this environment where there is no institutional constraint on the bed for real patient, one can provide the ground for the more efficient use of them in clinical environments in the future through creating readiness and empowerment in students. However, the existing educational facilities should be employed efficiently to develop capability. Adequate time and session should be allocated for training and work in laboratory. The quantity of students should be suitable for active participation by all of them and there should be continuous and routine program for presentation of education in all midwifery skills and frequent exercise. 

Training in laboratory of competencies should be taken as serious and the fixed and capable teachers should be employed for this purpose. Likewise, the continuous and accurate evaluation should be implemented on acquisition of the trained skills. Overlooking the efficient education in power laboratories and lack of preparation of students for presence in clinical environments in the future will be followed by adverse consequences and acts as impediment factor against student to achieve capability. 

**Access to information sources**

The free and easy access of students to internet and other numerous information sources is an efficient factor in their achievement of the needed capabilities. Referring to these information sources upon exposure to various problems in clinical environments may prepare the ground for retained learning. “We have free wireless system in dormitory. We can have access to resources whenever we like and this is very favorable”. (Participant student) 

Alternately, lack of comprehensive reference book in some of educational fields, shortage of new edition of textbooks in laboratory, and or postponement in introducing new edition of books pave the way for difficult access to information resources and acquisition of the needed capabilities. 

**Information **

**Awareness of educational goals **

The educational objectives lead to doing work in clinical environment for both the teacher and the student and their efficient role in making education and learning purposeful in clinical environment and developing of empowerment in students. “Presentation of educational goals is very favorable because both student knows what she wants in this unit and also knows what she should do and at the same time instructor knows what she ask from student”. (Participant teacher) On the other hand, overlooking educational goals that may be due to several factors such as lack of familiarity and acquaintance of faculty and student with them, lack of executive potential for some of goals, inadequate clinical cases, and ignoring unfulfilled goals in clinical environment are impediment factor against acquisition of empowerment. 

**Awareness of legal and legitimate issues**

Awareness of legal and lawful issues in clinical environment pave the way for creating motive and effort for empowerment in diagnosis and decision making. This familiarity should start gradually from time of attendance of students in clinical environment at lower semesters. Lack of knowledge about these regulations may cause lack of sensitivity of students to importance of legal and legitimate aspects of clinical measures and consequently lack of effort to acquire further capability to prevent from involvement in legal and occupational issues: “A midwife even though she is a student should know the legal and legitimate issues within the range of her work” (Participant midwife) 

## Discussion 

This study has explained the facilitators and impediment factors against empowerment of midwifery students in pregnancy and delivery care from viewpoint of students, teachers, and the employed midwives by qualitative method and based on framework of Kanter’s theory for the first time. The findings showed that one of the efficient and facilitator factors for acquisition of empowerment in pregnancy and delivery care is to support from student in clinical environments done by clinical instructors, the employed midwives, and classmates. The support factor has been also proposed as a key element of the results of other studies. The supportive environment for clinical learning may increase empowerment among students [**24**]. The positive supportive environment noticeably affects on their empowerment through creating sense of value in student as a learner, a member of the team, and as an individual [**25**]. The supportive climate has provided unique learning opportunities to acquire skill and knowledge and highly affected on professional socialization of students. Among them, the supportive role is very prominent in clinical instructors who are eager and motivated for training as well as giving care to the patient and also excellent communication skills, knowledge, and adequate clinical competencies and educational skills [**26**].

The findings in present study also indicated that the capable instructors possess professionally and educationally positive personality traits and they could establish positive relations with other individuals in clinical environment and they might play efficient role in empowerment of students through guiding and presentation of feedback and support. Also according to attitude of Japanese resident physicians, proposing adequate support is one of the foremost characteristics of a clinical teacher. Giving the student the chance for thinking, offering feedback to the student, and identifying those fields that require for improvement is deemed as important attributes of a good clinical teacher [**27**]. Giving chance to learner to discuss, knowledge and capability of instructor to convey educational items, and skills of clinical instructors effect on clinical training [**28**]. Professional competency and relationship among instructor and students are assumed as foremost characteristics of efficient clinical instructor [**29**]. The educational course should be held for empowerment and further readiness of clinical instructors in developing capabilities of students further in supportive climate and training of students should be delegated to the expert clinical instructors with adequate interest and motive. 

Findings showed that if the active personnel had the professional competency and positive relations and interactions with students, the employed staff could play an active role in their empowerment through the preparation of supportive climate. Creation of appropriate sphere, giving opportunity, encouraging students for active participation in decision-making process, discussion, and dialogue between employed midwives with them about already-taken clinical decisions may prepare the ground for learning and empowerment in clinical decision-making [**30**]. The positive relationships between personnel and students in clinical environments and also their updated information are also considered as efficient factors in clinical training of students [**31**]. In acquisition of learning experiences, role of the employed midwives is more important than model of student’s presence in clinical sites. The motivated midwives in the field of education with student and in midwifery profession may propose further support and training to students in clinical environments [**32**]. The midwives are introduced as role model for students in clinical settings and they can facilitate their learning by exposing students to learning experiences and supporting them under challenging conditions and increase their self-confidence [**33**]. The relations of employed midwife instructors with students substantially affect on their skills and confidence. The good midwives are available at required time and they explain the work for students and positively communicate with student [**34**]. Acceptance of person in clinical environment creates sense of attachment in the given individual. Under such conditions, student participates in activities and receives the appropriate support and guidance and takes the help and becomes capable [**35**]. The employed midwives play efficient role in acquisition of this sense of attachment and belonging to environment and its resultant empowerment [**36**]. To strengthen supportive role of personnel in educational hospitals in developing the needed capabilities in midwifery students the staff with scientific, professional, and ethical competency should be elected. These staff should be coordinated further with educational programs and acquire the necessary skills for training of students. 

In our findings, support of classmates from each other was also one of the facilitator factors in achieving of capability. In clinical environments, peers are deemed as a source of spiritual support and also support from social loneliness and play role [**37**]. Asking questions freely from each other along with positive interactions with other students in clinical environments is followed by sense of self-confidence, potential for achievement of success, self-efficacy, and reduced anxiety [**38**]. Learning beside a peer provides more positive learning experiences. Assessment of performance is improved both in trainer and learner and it is accompanied with satisfaction [**39**]. Through identifying individual requirements of teacher and student both in educational and social dimensions, instruction by peer is followed by positive outcomes [**40**]. To prepare a more supportive platform in clinical environments, employing of peer should be more noticed in educational strategies and planning. 

Access to clinical settings with appropriate climate, facilities, and conditions and continuous presence in these sites was one of the necessary sources and as one of the useful factors in access to empowerment in our study. Attendance in clinical environments along with various working backgrounds contributes to improving their capability through increase in experience of students [**41**]. Variation of different clinical conditions and quality of experiences in midwifery students in these settings may also affect on making professional decisions for them in future [**42**]. However, inadequate facilities and equipment in clinical settings restrict acquiring favorable experiences [**31**]. Short period of presence in clinical environments and their frequency and challenges caused by these frequent displacements are also considered as negative active factors in sense of empowerment of students [**43**]. Short-term presence in clinical sites restricts acquisition of skill for student and leads to shortage of opportunity for accurate assessment of student by instructor in clinical environment [**44**]. To acquire the needed capabilities for students, this point should be especially noticed that to use appropriate clinical environments with the required facilities and conditions for efficient training and also allocation of the needed time to attendance in this environment. 

The findings of the present study showed that as a valuable source, the laboratory of clinical skills has important position for students to achieve the needed capabilities. If the efficient education is presented in laboratory of competencies with accurate valuation of the given trainings, one can expect from the students to be prepared and capable of further and better attendance in real clinical environments and confrontation to the related challenging situations. 

In study of Carolan-Olah and Kruger, 2014], experiences of midwifery superior students indicated that they also preferred to emphasize more on clinical skills and time of exercise in laboratory. At the same time, they asked for acquiring further readiness in midwifery urgencies in laboratory [**45**]. When it is impossible for gaining experience for doing a task and or clinical experiences are not favorable, an exercise in the laboratory of skills may contribute to students to overcome to functions [**33**]. Presence in laboratory of expertise and simulation of situations through strengthening self-confidence, facilitation of learning and improvement of capabilities in person may play efficient role in readiness for clinical work [**46**]. Acquisition of experience and skill in work under midwifery urgency situations, simulation of unexpected events and clinical consequences caused by errors along with delegation of full responsibility of doing tasks to student in these simulated conditions prepare the ground for efficient and effective education and training in laboratory of skills and achieving the needed capabilities for students in midwifery task. 

In our study, ease of access to several and updated information resources such as books from library, internet, and electronic resources are deemed as facilitator factors for learning in clinical environment and access to empowerment that have been implied in other studies as well [**28**,**47**]. The educational goals and legal and legitimate issues in midwifery profession are some information that the students need to make the effort for empowerment and to achieve the required capabilities for efficient performance in clinical environment. 

## Conclusion

The presence of supportive climate for midwifery students in clinical settings plays an essential role in their achievement of the necessary capabilities for pregnancy and obstetric care. This support should be proposed comprehensively by instructors, personnel, physicians, and peers. Preparation of appropriate clinical environments and their equipment along with paying due attention to the laboratory of clinical skills may provide the necessary opportunities to acquire clinical experiences and empowerment for them. Formulation and presentation of clear, explicit, and accessible educational goals and dealing with legal and legitimate issues in theoretical and practical courses may also create the needed motives to make efforts for empowerment by clarifying the path of this trend. 
